# Measles outbreak investigation in Ginnir district of Bale zone, Oromia region, Southeast Ethiopia, May 2019

**DOI:** 10.11604/pamj.2020.36.20.21169

**Published:** 2020-05-14

**Authors:** Falaho Sani Kalil, Desta Hiko Gemeda, Mohammed Hasen Bedaso, Shukri Kabeta Wario

**Affiliations:** 1 Field Epidemiology Training Program, Department of Epidemiology, Jimma University, Jimma, Ethiopia; 2 Department of Epidemiology, Faculty of Public Health, Jimma University, Jimma, Ethiopia; 3 Field Epidemiology, Public Health Emergency Management, Bale Zonal Health Office, Bale Zone, Robe, Ethiopia

**Keywords:** Measles, outbreak, case control, Southeast Ethiopia, Bale zone

## Abstract

**Introduction:**

Measles is a vaccine-preventable viral infection of humans, primarily affecting children <5 years. During early 2019, outbreak of measles occurred in Ginnir district of Bale zone, Southeast Ethiopia. We investigated to describe the outbreak and identify risk factors.

**Methods:**

We conducted a descriptive and 1:2 unmatched case-control study in Ginnir district from March 18 to April 29, 2019. Fifty-six cases and 112 controls were recruited. For descriptive study, we identified 1043 cases recorded on the line-list and for case-control study, cases were identified using national standard case-definition. Mothers of case-patients and controls were interviewed using structured questionnaire. We estimated vaccine efficacy (VE) from case-control study. We conducted bivariate and multivariable logistic regression.

**Results:**

In four-months period, a total of 1,043 suspected measles cases epidemiologically linked to five laboratory confirmed cases reported. Of which, 555 (53.2%) were males and 714 (68.5%) were <5 years. The median age of cases was 36 months (IQR=12-60 months). The overall attack rate (AR) was 63/10,000 population with case fatality ratio of 0.5% (5 deaths/1043). Infant <9 months were the most affected age groups (AR=31/1000). Majority (79%) of measles cases were not vaccinated against measles. Last-year (2017/18) administrative measle vaccine coverage of the district was 76.7%. Being unvaccinated against measles (AOR=5.4, 95%CI=2.2-13.4), travel history (AOR=4.02, 95%CI=1.2-13.6), contact with measles case-patient (AOR=5.6, 95%CI=2.12-14.4) and mothers knowledge of measles transmission (AOR=0.36, 95%CI=0.15-0.87) were associated with measles infection. VE in children aged 9-59 months was 90% (95%CI=69-97%).

**Conclusion:**

This confirmed measles outbreak was caused by failure to vaccinate, as indicated by the high VE, low administrative coverage, and 79% unvaccinated cases. Strengthening routine and supplementary immunization are required.

## Introduction

Measles is an acute, highly contagious viral disease caused by measles virus in the family Paramyxovirus, genus Morbillivirus, transmitted primarily by respiratory droplets or airborne spray from the coughs and sneezes of person infected with the disease to mucous membranes in the upper respiratory tract or the conjunctiva [1),^2^]. Measles infection can result in serious complications such as blindness, encephalitis, or severe respiratory infections such as pneumonia [1-3]. The case fatality from measles is estimated to be 3-5% in developing countries [1),^2^]. Even though a safe and cost-effective vaccine is available, there were 229,068 measles cases reported in 2018 [4]. According to WHO report, measles infections increased in all regions of the world in 2019. Global cases of measles in the first three months of 2019 has quadrupled compared with the same time last year, Africa had witnessed the most dramatic rise - up 700%. In total, 170 countries reported 112,163 measles cases to WHO, in comparison to 28,124 cases across 163 countries during the same period in 2018 [5),^6^]. Measles incidence is still high in Ethiopia, above 50 cases/1,000,000 population per year [7]. Measles is endemic in Ethiopia with outbreak reported every year. During the year between 2016 and 2018, a total of 7445 confirmed measles cases were reported from Ethiopia [8]. Till August 2019, a total of 8,202 suspected measles cases were reported from four region; Oromia (4611 cases), Amhara (703 cases), Afar (548 cases) and Somali region (2,340 cases) [9]. Bale zone is one of zones in Oromia region which is extensively affected by measles outbreak and more than third (16/21) of woredas under this zone is affected [10]. Measles is one of the leading causes of death among children globally, particularly in developing countries. Approximately 110,000 measles death occurred globally in 2017-mostly children under the age of 5 years [5]. Economic impact studies of measles outbreaks in high-income countries illustrate a high cost of measles outbreaks and response activities [11]. According to evaluation of economic costs of a measles outbreak and response activities in Keffa Zone of Ethiopia, the economic cost of the outbreak and response was 758,869 United States dollars (US$) and household economic cost was US$29.18/case [12]. Outbreak preparedness and response is one of the five core strategies in the 2012-2020 WHO strategic plans for global measles and rubella [13]. The Africa Region as well as Ethiopia is working towards measles elimination by 2020. Ethiopia adopted these regional goals and strategies and has been taking important steps to control and ultimately to eliminate measles by 2020 [1]. Immunization services have been delivered through static and outreach sites nationwide since establishment of national immunization programme in 1980. The current routine immunization schedules recommend a dose of measles vaccination at 9 months of age [1]. Recently, Ethiopia launched measles vaccine second dose (MCV2) vaccination into the routine immunization programme in the second year of life on February 11, 2019 [7]. Despite all these efforts WHO-UNICEF coverage, estimate of measles first dose (MCV1) coverage in Ethiopia was 56% in 2010 and 57% in 2011 [14]. Likewise, the WHO-UNICEF estimates for measles vaccination coverage (MCV1) of Ethiopia in the years 2017 and 2018 indicate 65% and 61% respectively, while the administrative coverage was 92% in the year 2017 [7]. Measles outbreaks occur when the accumulated number of susceptible individuals is greater than the critical number of susceptible individuals, or epidemic threshold for a given population [3]. Various literature on outbreak investigation showed that the possible factors for measles infections were being unvaccinated for measles, low immunization coverage, malnutrition, poor cold chain management and travel history to measle area, presence of measles case in neighbor or in the household and having contact with person with measles case [15-18]. Despite the increase in immunization coverage (administrative) of measles in the country, there was a widespread measles outbreak reported from different zones of Oromia region including Bale zone in 2019. After having received this report from Bale zonal health department, an organized team that consists Field Epidemiology Training Program (FETP) residents was deployed to this woreda and investigated the outbreak. The aim of this study was to investigate the magnitude of measles outbreak and identify factors associated with measles infection in Ginnir district, Bale Zone, Oromia region, Southeast Ethiopia, 2019.

## Methods

**Study area and period**: Ginnir district is one of the 20 districts of Bale zone, Oromia region, Ethiopia. It is located at 557 km Southeast to Addis Ababa, the capital city of Ethiopia. The district has a total population of 164,702 in 2019. Of whom 51% (83,998) were females and 49% (80,704) were males. It has 32 rural kebeles (lowest administrative level), eight health centers and 32 health posts. Health centers in the district provide static immunization service while health posts provide an outreach service. Data for descriptive study were collected from December 15, 2018 to April 9, 2019. Case-control study data were collected from March 5-16, 2019 for cases and from May 15-26, 2019 for controls.

**Study design and sample size**: we used descriptive cross-sectional study to describe magnitude of measles outbreak and a 1:2 unmatched case-control study design to identify the risk factors associated with measles infection. All suspected measles cases recorded on the line-list were included for descriptive study. For case-control study, a total of 168 samples (56 cases and 112 controls) were included. We found the cases listed on a line-list and then, searched to their house for an interview. Two controls from the same household or neighborhood who resides in the same kebele with cases were recruited for each selected case.

### Case definitions

**Suspected measles case**: any person with fever and maculopapular (non-vesicular) generalized rash and cough, coryza or conjunctivitis (red eyes) [1].

**Confirmed measles case**: a suspected case with laboratory confirmation (positive IgM antibody) or epidemiologically linked to confirmed cases [1].

**Measles death**: any death from an illness that occurs in a confirmed case or epidemiologically linked case of measles within one month of the onset of rash [1].

### Enrolment of cases and controls

**Cases**: residents of Ginnir district that have clinical signs and symptoms of measles based on the case definitions in the national measles guideline that were either laboratory confirmed or epidemiologically linked to confirmed cases.

**Controls**: residents of Ginnir district who reside in the same household or neighborhood to a case and who did not fulfill measles case definitions.

**Data collection procedures**: for descriptive study, we identified the line-listed measles cases. For case-control study, cases were identified using national standard case-definition and we found the line-listed cases and then, searched to their house for an interview. Controls who reside in the same kebeles with cases were identified from non-cases after outbreak ended. A structured questionnaire was used to collect information on socio-demographic characteristics and risk factors for measles infection. Parents/caregivers were interviewed using structured questionnaire on their children's behalf. Vaccination history was assessed by documented vaccination status whenever vaccination card is available or based on the recall of their caregivers when the card is unavailable. Accumulation of susceptible population was assessed using five years administrative routine measles vaccination coverage and supplementary immunization activities (SIAs) coverage of the district. Data regarding vaccination coverage and cold chain management were reviewed and collected from the Ginnir district health office.

Vaccine effectiveness: we estimated vaccine effectiveness (VE) from case-control study using the following formula [3,19,20]. VE= 1-RRProtective≈1-ORProtective; VE = [1- AOR vaccinated Vs unvaccinated] x 100. Where, RRProtective is the protective relative risk associated with vaccination, which can be approximated by the protective odds ratio (ORProtective), in rare diseases such as measles [19),^20^]. We estimated VE in children aged 9-59 months because routine measles vaccination in Ethiopia starts at 9 months of age and this outbreak mainly affected children aged ≤59 months.

**Data analysis procedures**: for descriptive study, line list data were cleaned and then, analyzed by Microsoft Excel 2016 using pivot table. Descriptive analysis on measles cases were computed and summarized by person, time, and place. Distribution of measles case by kebele were summarized by spot map using Arc GIS version 10.3. Case control study data were checked for completeness and consistencies, and then coded and entered using Epi-data version 4.4, and then exported to SPSS version 23 for analysis. All categorical variables were cross-tabulated with outcome variable, and described by their frequencies and proportion for cases and controls groups. All explanatory variables that were significantly associated with the outcome variable in the bivariate logistic regression at p< 0.20 were entered into multivariable logistic regression model using backward elimination stepwise method to identify independent factors associated with measles infection. The model adequacy was checked using Hosmer and Lemeshow goodness-of-fit test and Omnibus test. Adjusted odds ratio (AORs) with their corresponding confidence intervals (CIs) were used to assess the strength of associations between the outcome and predictor variables at P-value <0.05. Results were presented by text, table, figure and map.

**Ethical consideration**: support letter to conduct the study was written from Jimma University and Bale zonal health department. We obtained permission letter from Ginnir district health office. The participants parents/caregivers were informed about the objectives of study and their willingness to participate. Verbal informed consent was obtained from all study participant’s parents/caregivers and confidentiality was assured.

## Results

### Descriptive epidemiology

**Figure 1:**
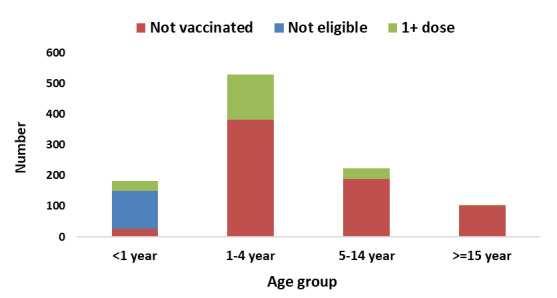
Vaccination status of measles cases in Ginnir district by age group, Bale zone, Oromia region, Southeast Ethiopia, 2019

**Description of measles cases by person**: from December 12, 2018 to April 9, 2019, a total of 1,043 suspected measles cases epidemiologically linked to confirmed cases were reported from Ginnir district. Of these, 5 (0.48%) cases were confirmed by laboratory investigation (IgM positive). More than half of measles cases 555 (53.2%) occurred in males and 714 (68.5%) of cases occurred in children below five years. The median age (IQR) of cases was 36 months (12-60 months). The overall attack rate (AR) was 63/10,000 population with case fatality rate of 0.5% (5 deaths/1043). The age-specific attack rates vary and higher in children below <9 months (31/1000 populations). The AR in male and female were 6.6/1000 population and 6.1/1000 population respectively (Table 1). Regarding vaccination status, more than three-fourth of measles cases 824 (79%) were not vaccinated against measles while 213 (20.4%) of the cases received only one dose and two (0.2%) of the cases received two or more doses of measles vaccine. Among 182 measle cases which occurred in infants, 150 (82.4%) were not vaccinated against measles. Of these infants unvaccinated against measles, 27 (18%) were eligible for vaccination (9-11 months), whereas 123 (82%) infants were not eligible for vaccination (<9 months) (Figure 1).

**Table 1:** Measles attack rate and case fatality rate by age group in Ginnir district of Bale zone, Oromia region, Southeast Ethiopia, May 2019

Age group	Total population	Number of cases	Number of deaths	Attack Rate (per 1000 population)	Case Fatality Rate (%)
<9 months	3977	123	0	31	0.0
9 month-4 year	23083	591	3	25.6	0.5
5-14 year	51337	224	2	4.4	0.9
>=15 year	86304	105	0	1.2	0.0
**Total**	164702	1043	5	6.3	0.5

**Figure 2:**
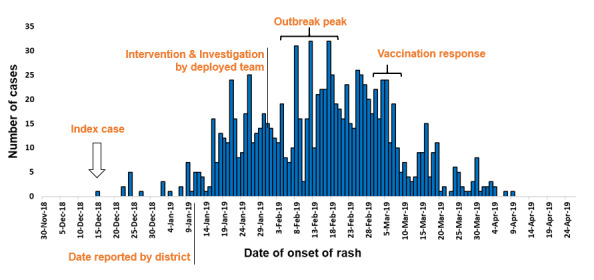
Epidemic curve showing number of measles cases and date of onset of rash in Ginnir district, Bale zone, Oromia region, Southeast Ethiopia, 2019

**Description of measles cases by time**: the outbreak which lasted for 117 days started in the 50^th^ WHO epidemiologic week of 2018 and ended in the 15^th^ week of 2019. The epi-curve shows several peaks and suggesting a propagated outbreak (Figure 2). The index case of this outbreak was a 29 years old man from Harawa Misra kebele of the district, who was seen at the health facility on December 19, 2018 and date of onset of rash on December 15, 2018. The man travelled to adjacent district (Sawena) of Ginnir district to attend funeral ceremony two weeks before developing rash and, there was measles outbreak in Sawena district at a time. After one incubation period, the first outbreak occurred with 5 suspected measles cases occurring among residents of the same village with index case and they visited the same health facility on December 28, 2018 with date of onset of rash on December 24, 2018. After one incubation period, the second outbreak occurred in the first week of January 2019 and there was gradual increase in the number measles cases starting from the second week of January 2019 (2^nd^ WHO Epi week) (Figure 2).

**Description of measles cases by place**: all 32 kebeles of Ginnir district were affected by this outbreak. Nearly two-third of cases occurred in four kebeles of the district, which are hard to reach kebele to provide immunization service and supervision during rainy season due to inaccessible road. The AR varies among the kebeles of district with the highest attack rate of (35.5 per 1000 population) reported from Getara kebele and followed by Harewa-misra (33.2 per 1000 population), Guberbaja (13.1 per 1000 population) and Suragefate kebele (12.7 per 1000 population). These four kebeles were the remote kebeles of the district with many hours walk on foot during rainy season, which makes it difficult to provide routine immunization service. The finding from spot map indicate clusters of measles cases in Getera and Harewa-misra kebele of the districts, which are the kebeles with high attack rate and hard to reach.

**Vaccination coverage and pool of susceptible population**: the administrative coverage of measles vaccination in the district from 2014 to 2017 ranges from 77% to 98% with last year (2017/18) coverage of 76.7%. The most recent supplementary immunization activities (SIAs) targeting children of 6-59 months was conducted in February 2017 with administrative coverage of above 100%. The routine measles vaccination coverage in hard to reach kebeles in 2018 were 77% in Getara, 44.5% in Harewa-misra, 52.9% Suragefete and 61.3% Guberbaja kebeles. Out of 32 health posts found in Ginnir district, refrigerator for cold chain management is available only in eight health posts and all available refrigerators are unfunctional. The outbreak response immunization targeting children of 6 months to 14 years was initiated in the 11^th^ week of the epidemic (the first week of March 2019) when the epidemic curve started to decline and after the cases reached 778 (75%). According to district health office report, 98% administrative coverage was achieved. The finding of accumulation of susceptible population from 2014/15-2018/19, which is 3750 were greater than 2/3^rd^ of the annual birth cohort in 2019/20 (3713) (Additional file 1).

**Case-control study**: a total of 56 cases and 112 neighborhood controls participated in the study to identify the risk factors for measles infection in Ginnir district. Of the total participants, 32 (51.7%) of cases and 61 (54.4%) of controls were males. The median age (IQR) of the cases and controls were 24 months (IQR=11-48 months) and 36 months (IQR=12-60 months) respectively. The majority of both cases 32 (57.1%) and controls 63 (56.2%) were in the age group of 1-4 years. Forty-two (80.4%) of cases and 97 (86.8%) of controls caregivers had no formal education (Table 2). The clinical presentation of case-patients was consistent with measles infection, with all cases having history of rash and fever, 55 (98.2%) had cough, 50 (89.3%) had conjunctivitis and 51 (91.1%) had coryza. During bivariate analysis eight variables were selected as candidate for the final model (Table 2).

**Table 2:** Bivariate analysis of sociodemographic factors for measles infection in Ginnir district, Bale zone, Oromia region, Southeast Ethiopia, May 2019

	Cases No. (%)	Controls No. (%)	COR (95%CI)	P-Value
**Sex**				
Male	32 (57.1)	61 (54.5)	1	
Female	24 (42.9)	51 (45.5)	0.9 (0.47-1.7)	0.742
**Age group**				
<1 year	14 (25.0)	19 (17)	1	
1-4 year	32 (57.1)	63 (56.2)	0.69 (0.31-1.6)	0.369
5-14 year	10 (17.9)	30 (26.8)	0.45 (0.17-1.2)	0.118*
**Marital status of caregiver**				
Single	6 (10.7)	15 (13.4)	1	
Married	47 (83.9)	95 (84.8)	1.24 (0.45-3.4)	0.68
Othersa	3 (5.4)	2 (1.8)	3.75 (0.49-28.4)	0.201
**Ethnic group**				
Oromo	49 (87.5)	102 (91.1)	1	
Amhara	5 (8.9)	6 (5.4)	1.74 (0.51-5.96)	0.382
Somali	2 (3.6)	4 (3.6)	1.04 (1.8-5.9)	0.964
**Religion**				
Muslim	53 (94.6)	106 (94.6)	1.0 (0.21-4.16)	1.00
Orthodox	3 (5.4)	6 (5.4)	1	
**Educational status of caregiver**				
No formal education	45 (80.4)	97 (86.6)	1	
Primary	5 (8.9)	8 (7.1)	1.35 (0.42-4.35)	0.618
Secondary and above	6 (10.7)	7 (6.3)	1.85 (0.59-5.8)	0.294
**Occupation of caregiver**				
Housewife	36 (64.3)	87 (77.7)	1	
Daily laborer	8 (14.3)	7 (6.3)	2.76 (0.93-8.2)	0.067*
Merchant	9 (16.1)	14 (12.5)	1.6 (0.62-3.9)	0.350
Othersb	3 (5.4)	4 (3.6)	1.8 (0.39-8.5)	0.451
**Family size**				
<5Person/HH	13 (23.2)	34 (30.4)	1	
>=5Person/HH	43 (76.8)	78 (69.6)	1.44 (0.69 - 3.02)	0.33

a Divorced and widowed, b Student and government employee, HH=Household

* Significant at p-value of <0.20 in bivariate analysis

**Independent risk factors independently associated with measles**: after adjusting for confounding effect in the multivariable regression model, four variables were found to be significantly associated with measles infection. Being unvaccinated against measles was found to be an independent risk factor for having measles. Child who hadn't vaccinated against measle vaccine was five-fold increased odd of having measles as compared to vaccinated (AOR=5.4, 95% CI: (2.2-13.4)). Similarly, individual who had travel history to the area with active measles was four times more likely to have measles compared to those who hadn't traveled (AOR=4.02, 95% CI: (1.2-13.6)). Having contact with measles patient was an independent risk factor for measles infection. The odd of having measles among individuals who had contact with measles case-patient was almost six-fold higher when compared to those who had no contact history (AOR=5.6, 95% CI: (2.12-14.4)). In addition, knowledge of caregivers on mode of transmission of measle were found to be protective for having measles infection. Individuals whose caregivers knew measles mode of transmission were 64% less likely to have measles compared to those individuals whom caregivers lack knowledge about mode of transmission (AOR=0.36, 95% CI: (0.15-0.87)) (Table 3).

**Table 3:** Factors independently associated with measles infection in Ginnir district, Bale zone, Oromia region, Southeast Ethiopia, May 2019

Characteristics	Cases No. (%)	Controls No. (%)	COR (95%CI)	P-Value
**Vaccination status**				
No	40 (71.4)	38 (33.9)	6.05 (2.84-12.9)	<0.0001*
Yes	12 (21.4)	69 (61.6)	1	
Unknown	4 (7.1)	5 (4.5)	4.6 (1.1-19.6)	0.039
**Travel history to the area with active measles**				
No	35 (62.5)	105 (93.8)	1	
Yes	21 (37.5)	7(6.3)	9.0 (3.53-22.97)	<0.0001*
**Contact with measles patient**				
No	21 (37.5)	97 (86.6)	1	
Yes	35 (62.5)	15 (13.4)	10.7 (5.01-23.21)	<0.0001*
**Measle case presence in home**				
No	43 (76.8)	100 (89.3)	1	
Yes	13 (23.2)	12 (10.7)	2.5 (1.06-5.97)	0.036*
**Knowledge of measles transmission**				
No	44 (78.6)	55 (49.1)	1	
Yes	12 (21.4)	57 (50.9)	0.26 (0.13-0.55)	0.0003*
**Knowledge of measles is vaccine preventable**				
No	40 (71.4)	65 (58)	1.8 (0.91-3.61)	0.093*
Yes	16 (28.6)	47 (42)	1	
**Malnutrition status**				
Normal	38 (67.9)	82 (73.2)	1	
Moderate	13 (23.2)	25 (22.3)	1.12 (0.52-2.4))	0.770
Severe	5 (8.9)	5 (4.5)	2.2 (0.59-7.9)	0.245
**Time taken to reach health facility**				
<30 minutes	29 (51.8)	48 (42.9)	1	
30-60 minutes	20 (35.7)	48 (42.9)	0.69 (0.34-1.38)	0.295
>60 minutes	7 (12.5)	16 (14.3)	0.72 (0.27-1.97)	0.527

* Significant at p-value of <0.20 in bivariate analysis

**Measles vaccine effectiveness**: when the case-control investigation data were analyzed for children aged 9-59 months, 75% (42/56) of case-persons compared to 62.5% (70/112) of controls had a history of measles vaccination (AOR = 0.10; 95% CI: 0.03-0.31). Using this information, the estimated measles vaccine effectiveness was 90% (VE= 90%; (95% CI: 69-97%) in children aged 9-59 months).*’*

## Discussion

This study focused on describing the magnitude of measles outbreak and identify risk factors for measles infection in Ginnir district. The measles disease was confirmed after five serum samples were positive for measles specific-IgM antibodies and other cases were epidemiologically linked to the laboratory-confirmed cases. Our investigation showed that a prolonged measles outbreak occurred in Ginnir district lasted for 117 days. The finding of descriptive study shows the measles outbreak affected a wider age range from 2 months to 38 years with overall attack rate of 63 cases/10000 population. We found higher attack rate than the attack rate of measles outbreak investigation conducted in Guji zone of Ethiopia (81/100000), including the study conducted in Simada district of South Gondar Zone (41/100000) and Kabridehar district of Somali Regional State, Ethiopia reported AR of 4/10000 [16,21,22]. Similarly, our finding is higher than the studies conducted in developing countries; in Cameroon (34/100000) and in China (3.3/100000) [17),^23^]. However, our finding is comparable with the study conducted in Kebridehar Town of Ethiopia (79/10000) [15]. The higher attack rate is possibly due to accumulation of susceptible population which may have contributed to the spread of the disease faster than expected. Furthermore, during the last two years, there was poor vaccination coverage in the district (77% and 78%) and hard to reach kebeles of the district that are difficult provide routine immunization also contributed for high attack rate of measles. The overall high attack rates with wider age range might suggest the persistent low routine immunization coverage over several years, that may have led to the current outbreak. The implication of this finding is a need to strengthen routine immunization and SIAs, monitoring of accumulation of susceptible individuals to protect both target and non-target age groups.

In addition, a relatively large proportion of cases in <9 months (123/182 infants, 67.7%) were affected by the outbreak. This finding is in line with measles outbreak investigation report of the study conducted in Guji zone of Ethiopia, which indicate large proportion of measles cases in infants less than 9 months [21]. The highest attack rate of 31 cases per 1000 population was also observed among these children, that's the group not yet eligible for vaccination. This is a reflection of their vulnerability between 0-9 months, before receiving vaccine. This finding also creates a concern regarding to the age at which vaccination should start. The occurrence of measles cases in infants less than 9 months might be due to contributing factors like malnutrition, and it may also be that children of this age group may have no maternal antibody at the very beginning which may indicate a long-standing problem with measles vaccination [1,2,21]. Other important finding from descriptive study is case fatality rate (CFR). Our study found that the CFR from measles is 0.5% (5 deaths/1043) with the highest CFR of 0.9% among children aged 5-14 years. In Ethiopia, the expected case-fatality rate from measles is between 3% and 6% [1]. The case fatality rate from measles is estimated to be 3-5% in developing countries but may reach more than 10% during epidemics [2]. The CFR observed in our study was comparable to the findings of measles outbreak investigations conducted in Guji zone of Ethiopia (CFR=0.2%) [21] and in Bihar, India (CFR=0.78%) [24]. However, it is lower than the report of outbreak investigations conducted in Simada district of South Gonder zone, Ethiopia (CFR=13.4%) [22] and in Kebridehar town of Somali region, Ethiopia (1.8%) [15], Herena and Dawe-Serer districts of Bale Zone of Ethiopia (9.1%) [25]. But, the CFR in our investigation is higher than the finding of measles outbreak investigations in Kabridehar district of Somali region [16], Ethiopia and Nylon Health District of Cameroon; which reported no death [23]. The low case fatality rate in this study is possibly due to implementation of early response and case management in affected kebeles. It might be also due to the fact that death at the community level is not registered and the reported deaths includes only health facility deaths.

Result from multivariable logistic regression of case-control study showed that, being unvaccinated against measles, having travel history to the area with active measles, having contact with a person suspected to have measles, and knowledge of mother on the measles transmission were factors independently associated with measles infection. Being unvaccinated against measles is significantly associated with having measles. Child who hadn't vaccinated against measle vaccine had five-fold increased odd of having measles as compared to vaccinated. This is in line with the finding of case control study conducted in Kabridehar town and Kabridehar district of Somali Regional State, Ethiopia, indicating that being vaccinated against measle vaccine is protective factor for contracting measle [15),^16^]. Similarly, our finding is consistent with the case control study conducted in Uganda and China indicating unvaccinated against measles was responsible for most measles infection in children [18),^19^]. This is due to the fact that vaccination against measles is vital for prevention of measles infection [1-3]. To prevent measles outbreaks or interrupt transmission and to enhance elimination of measles, 95% population immunity is needed [1]. The administrative routine immunization coverage of measles in Ginnir district in 2017/18 (2010 E.C.) and the estimated measles vaccine coverage 76.7% and 65.7% respectively, which indicates suboptimal population immunity to prevent an outbreak [1),^2^]. This finding suggests that, the occurrence of measles outbreak is possibly due to build-up of susceptible persons for measles infection. Individual who had travel history to the area with active measles were four times more likely to have measles compared to those who hadn't traveled. This finding is supported by similar study conducted in Ilu-Ababora zone of Oromia, Ethiopia [26]. This is due to the high risk of getting infection in the area of active measle or due to contact with infected person. Having contact with measles patient were independent risk factor of contracting measle. The odd of having measles among individuals who had contact with measles case-patient was almost six-fold higher when compared to those who had no contact history. This finding is supported by the outbreak investigation findings of case control study conducted in Kabridehar town [15] and Kebridehar district of Somali region of Ethiopia [16], and other similar study done in Zimbabwe [27], indicating having contact with measles case were more than threefold increased odd of having measles. This is due to the nature of measles transmission which is by respiratory droplets or by direct or indirect contact with nasal and throat secretions of infected persons and the secondary attack rate of measles is above 90% in the presence of susceptible individuals [1,2,28]. When measles virus is introduced to a non-immune population, nearly 100% of individuals will become infected and develop clinical illness [1].

Knowledge of mother/caregivers on measles transmission was found to be protective for measles infection. Individual whose mother/caregivers knew measles mode of transmission were 64% less likely to have measles compared to those individuals whom caregivers lack knowledge about measles. The finding is consistent with the study conducted in Bassona Worena Woreda of Amhara region, Ethiopia indicating that mothers who were aware of measles immunization importance were three times more likely to immunize their children of mothers who had poor knowledge on measles vaccine [29]. Similarly, our finding is supported by the study conducted in Northern and Southern part of Ethiopia, which reported some households with poor knowledge have poor health seeking behavior for measle cases and think the modern medicine aggravates the diseases [30),^31^]. Moreover, the study conducted in Southwest Ethiopia revealed that mothers who had adequate knowledge of measle infection have less risk of acquiring measles [32]. The estimated vaccine efficacy from our investigation for children aged 9-59 months, was 90% (95% CI: 69-97%). According to WHO, in countries where the first dose is given at 9 months, vaccine efficacy is estimated to be approximately 85% [3]. The seroconversion rate of measles vaccine at 9 months of age is 85% [1]. Our finding is consistent with the study conducted in Southwest Ethiopia, indicating VE= 90%, 95% CI: 83-91% [32], and other studies for one-dose measles vaccination reported VE of 85-94% [33]. High vaccine efficacy and low administrative vaccination coverage suggests that a failure to vaccination. Our study had multiple limitations. First, majority of participants lacked immunization cards. We asked the participant or/and caretakers whether their children had received a measles vaccine at age 9 months on the upper left arm. Recall bias is likely due to absence of vaccination card that was difficult to determine the vaccination status and exact date of vaccination. Second, there might be recall bias on the date of rash onset by the participant and their caregivers. Third, potential inclusion of false positive cases (i.e. not meeting case definition) as line list was collected by health workers at a lower level. Fourth, only health facility deaths were recorded.

**Prevention and control measures taken**: cases were treated to prevent further spread and reduce morbidity and mortality attributed to measles using medications (antibiotic, oral rehydration salt, tetracycline eye ointment, vitamin A). Active measles case search and management were conducted in all affected kebeles of the district. The line list was updated with the newly identified cases during active case search. Health education and community mobilization activities were conducted on measles prevention and control measures. In addition, mass vaccination as part of outbreak response was provided for children from 6 months to 14 years.

## Conclusion

The current outbreak occurred in the remote pocket of the district where it is hard to reach area and then affected all kebele and adjacent districts. This outbreak affected a wider age range with high attack rate is observed. Majority of cases occurred in children below five years, with children below 9 months being mostly affected age group. More than three quarters of the cases of this outbreak were not vaccinated against measles. The administrative and estimated measles vaccine coverage was low and vaccine effectiveness was high. Factors that contributed for the occurrence measles infection include; being unvaccinated against measles, having travel history to the area of active measles, having contact with measle cases, and poor knowledge of caregivers on the measles transmission. This confirmed measles outbreak was caused by failure to vaccinate, as indicated by accumulation of susceptible individuals, low vaccination coverage, high vaccine effectiveness and 79% unvaccinated cases. Therefore, strengthening routine immunization and SIAs, monitoring of accumulation of susceptible individuals' overtime, enhancing the awareness of the community toward measles transmission and cold chain maintenance at health post level is required. In addition, accurate reporting of vaccination coverage is required to guide interventions.

### What is known about this topic

- Measles is endemic in Ethiopia with high incidence (50 cases/1,000,000) and outbreak reported every year;
- Measles is one of the leading causes of death among children globally, particularly in developing countries;
- Measles outbreaks occur when the accumulated number of susceptible individuals is greater than the critical number of susceptible individuals, or epidemic threshold for a given population.

### What this study adds

- Infant below 9 months were the most affected age groups (AR=31/1000) from the measles outbreak;
- More than three-fourth (79%) of measles cases were not vaccinated against measles;
- The confirmed measles outbreak was caused by failure to vaccinate, as indicated by the high vaccine effectiveness, low administrative coverage, and 79% unvaccinated cases.

## Competing interests

The authors declare no competing of interests.
